# The developmental origins of sex-biased expression in cardiac development

**DOI:** 10.1186/s13293-019-0259-1

**Published:** 2019-09-05

**Authors:** Daniel F. Deegan, Reza Karbalaei, Jozef Madzo, Rob J. Kulathinal, Nora Engel

**Affiliations:** 1 Fels Institute for Cancer Research, Lewis Katz School of Medicine, Temple University, 3400 N. Broad St, Philadelphia, PA 19140 USA; 2 Department of Biology, College of Science and Technology, Temple University, 1900 N. 12th St, Philadelphia, PA 19122 USA

**Keywords:** Sex chromosomes, Regulatory networks, Cardiogenesis, Sexual dimorphism, Differentiation

## Abstract

**Background:**

Expression patterns between males and females vary in every adult tissue, even in organs with no conspicuous dimorphisms such as the heart. While studies of male and female differences have traditionally focused on the influence of sex hormones, these do not account for all the differences at the molecular and epigenetic levels. We previously reported that a substantial number of genes were differentially expressed in male and female mouse embryonic stem (ES) cells and revealed dose-dependent enhancer activity in response to *Prdm14*, a key pluripotency factor expressed more highly in female ES cells. In this work, we investigated the role of *Prdm14* in establishing sex-specific gene expression networks. We surveyed the sex-specific landscape in early embryogenesis with special reference to cardiac development. We generated sex-specific co-expression networks from mouse ES cells, examined the presence of sex-specific chromatin domains, and analyzed previously published datasets from different developmental time points to characterize how sex-biased gene expression waxes and wanes to evaluate whether sex-biased networks are detectable throughout heart development.

**Results:**

We performed ChIP-seq on male and female mouse ES cells to determine differences in chromatin status. Our study reveals sex-biased histone modifications, underscoring the potential for the sex chromosome complement to prime the genome differently in early development with consequences for later expression biases. Upon differentiation of ES cells to cardiac precursors, we found sex-biased expression of key transcription and epigenetic factors, some of which persisted from the undifferentiated state. Using network analyses, we also found that *Prdm14* plays a prominent role in regulating a subset of dimorphic expression patterns. To determine whether sex-biased expression is present throughout cardiogenesis, we re-analyzed data from two published studies that sampled the transcriptomes of mouse hearts from 8.5 days post-coitum embryos to neonates and adults. We found sex-biased expression at every stage in heart development, and interestingly, identified a subset of genes that exhibit the same bias across multiple cardiogenic stages.

**Conclusions:**

Overall, our results support the existence of sexually dimorphic gene expression profiles and regulatory networks at every stage of cardiac development, some of which may be established in early embryogenesis and epigenetically perpetuated.

**Electronic supplementary material:**

The online version of this article (10.1186/s13293-019-0259-1) contains supplementary material, which is available to authorized users.

## Background

It has long been acknowledged that clinical presentation of cardiovascular disease differs between men and women. Even in healthy adults, there are baseline sex differences in cardiovascular structure and function [1]. With the advent of sensitive sequencing technologies, a surprising amount of transcriptional and epigenomic variability has recently been shown between men and women in most adult tissues, including the heart [2–5]. Detailed studies in cardiomyocytes in humans, rats, and mice have also revealed sexual dimorphisms in transcriptome and function [6]. Most of these differences have been attributed to hormonal factors, yet results from many studies have shown that pathways other than hormones play an important role [7, 8]. For example, sex chromosomes contribute independently to sex biases in gene expression, although the specific sex chromosome-linked genes and their downstream targets have not been elucidated.

Genetic and epigenetic factors involved in normal cardiac development have been extensively characterized [9–14] and the transcriptional networks vital for cardiogenesis are well established. Generally, there has been no expectation in the developmental field that sex is relevant to early embryonic processes. Yet many congenital heart defects exhibit sex biases in presentation, mortality, and morbidity [15] and are primarily due to disruptions occurring before gonad formation. Furthermore, gestational insults, such as maternal undernutrition, are associated with sex-specific alterations in fetal heart development [16]. These imbalances have not been explained at either the genetic or developmental level and indicate that sex is an important biological variable during early embryogenesis.

In fact, sex-specific expression differences in early embryogenesis are widespread across the animal kingdom. Recent studies in non-mammalian model organisms have reported sex-biased expression at stages in which visible phenotypic differences between the sexes have not yet become apparent [17]. However, the question of whether this is also true of mammals has rarely been addressed.

One exception is the growing body of reports on mouse embryonic stem (ES) cells, which are self-renewing, pluripotent derivatives from pre-implantation embryos. Understanding the gene networks that control ES cells has been a major focus for many years [18–20] and recently, a surprising amount of sexual dimorphism in gene expression has been revealed in ES cells in both mice and humans [21–24]. Some expression differences were expected due to the presence of two active X chromosomes in females versus one in male cells. However, the majority of biases emanate from autosomal genes, including genes encoding dose-dependent transcription factors (TFs) and epigenetic and remodeling enzymes (EREs). This suggests that sex-specific gene networks are established by the sex chromosomes before X chromosome inactivation (XCI) occurs in female cells. Sophisticated network analyses have provided insight into the biology of organ development and can be deployed on the available data to address this possibility.

Evidence supports that sex-biased expression of regulatory factors in early embryogenesis establishes sex-specific epigenomic landscapes. Yet whether these differences are reversed by dosage compensation or are perpetuated during embryogenesis, with consequences for organogenesis and beyond, is unknown. Thus, it is necessary to characterize male and female transcriptomes over ontogeny in mammalian systems and to determine if they are connected to later adult phenotypes.

Here, we propose that the sex-biased expression of certain TFs and EREs in early development marks the genome with lasting effects across the lifespan [25]. We posit that while lineage specification decreases the range of sex-biased gene expression, sex-specific epigenetic marks persist and result in differential expression at later developmental stages [25]. We characterize candidates for these effects by elucidating co-expression and protein-protein interaction networks underlying the sex biases in male and female mouse ES cells. Our results highlight a co-expression module that is highly correlated with sex chromosome composition and identifies *Prdm14*, a sex-biased gene with higher expression in female ES cells, as a key regulator of sex biases in ES cells. Using heart development as a model process, we report sex-biased expression in male and female ES cells differentiated to cardiac precursors, in in vivo embryonic hearts and in adult cardiomyocytes. By focusing on transcriptional and epigenetic factors, we identify a subset of sex differences established in early embryogenesis that persist throughout lineage determination and cardiac organogenesis. Furthermore, we find evidence that *Prdm14* regulates target genes that are sex-biased during cardiac development and, surprisingly, in the adult heart, when *Prdm14* is no longer expressed.

## Methods

### Construction of weighted gene co-expression network and modules

We used a previously published RNA-sequencing (RNA-seq) dataset from six male (40, XY) and six female (40, XX) mouse ES cell lines (GSE90516) for network analysis [24]. We derived these cell lines from independent F1 hybrid blastocysts resulting from reciprocal crosses of mouse substrains C57BL/6 and CAST/EiJ by natural mating. Each cell line was maintained in ES cell culture medium (DMEM, 15% fetal calf serum, 1 mM sodium pyruvate, 2 mM l-glutamine, 1% non-essential aminoacids, 0.1 mM 2-mercaptoethanol and 1000 U/ml leukemia inhibitory factor) in 5% CO_2_ at 37°. The data was generated using HiSeq 2500 single end reads of 50 base pairs. We reported hundreds of coding and non-coding RNAs that were differentially expressed between male and female ES cell lines, after filtering for strain-specific effects [24].

To prevent bias, all aligned transcripts were used to establish a weighted gene co-expression network analysis (WGCNA), a widely used systems biology method that employs gene expression data to construct a scale-free network [26]. The WGCNA package in R, version 1.6, is available at https://cran.r-project.org/web/packages/WGCNA/index.html. For the analysis herein, Pearson’s correlation matrices were calculated for all pairs of genes evaluating the correlation coefficient between gene m and gene n such that Smn = |cor(m,n)|. Next, the Pearson’s correlation matrices were transformed into matrices defining connection strengths using the power function a_mn_ = power(S_mn_,β) = |Smn|^β^. In doing so, strong correlations are emphasized and the influence of weak correlation is reduced on an exponential scale. To obtain a scale-free network, we performed network topology analysis for thresholding powers from 1 to 20. The lowest power value for scale-free topology was 10, so β was set at 10.

The connectivity of pairs of genes was evaluated by calculating topology overlap (TO). TO is a robust indicator of the relationships among neighborhoods of genes. The TO was then used to perform hierarchical average linkage clustering to identify gene co-expression modules. Modules are branches of a hierarchical cluster tree defined using the top-down dynamic tree cut method [27] with a minimum module size of 50 genes. After module identification, a *t* test was used to calculate the *p* value of candidate genes. The gene significance (GS) was defined as the mediated *p* value of each gene (GS = lgP). From this, the module significance (MS) was defined using the average GS from all the genes within said module.

### Transcription factor motif analysis

The set of genes within the module most highly correlated with cell sex, comprised of 1624 genes, was analyzed for known and de novo transcription factor motif binding sites. The parameters were set to cover the promoter using − 5000 to + 1000 bp of the transcriptional start site in the HOMER online software suite (http://homer.ucsd.edu/homer/) [28].

### Ingenuity pathway analysis

We analyzed gene sets using the ingenuity systems pathways analysis (IPA) tool (Qiagen; Redwood City, CA). Datasets were subjected to IPA Core Analysis and then analyzed using IPA Upstream Regulator, Downstream Effects, and Canonical Pathways analytic tools. To capture regulatory networks, we focused on transcription factors and epigenetic and remodeling enzymes. The IPA output was exported as Microsoft Excel files to prepare the supplemental tables.

### Chromatin immunoprecipitation and sequencing

Four low-passage (p7-9) independent mouse ES cell lines, two male lines (40, XY) and two female lines (40, XX), were grown on inactivated C57BL/6 mouse embryonic fibroblasts (MEFs). The MEFs are prepared from pooled embryos and include both male and female cells. ES cells were passaged at least twice prior to harvest to achieve high cell numbers for chromatin immunoprecipitation and sequencing (ChIP-seq). Cells were collected using 0.25% Trypsin + EDTA and MEF-depleted for 1 h at 37 °C in 5% carbon dioxide. After collecting the ES cells, residual MEFs were less than 1.5% of the final cell suspension. Because of their low numbers and the fact that they are a mixed population of male and female cells, any remaining MEFs are not expected to skew the results obtained from the ES cells. ES cells were cross-linked using formaldehyde at a final concentration of 1% followed by quenching with 1 M glycine. Sonication, immunoprecipitation, library construction, and sequencing were performed as previously described with minor modifications [29]. Briefly, three consecutive lysis buffers were used to ensure adequate nuclei release. Sonication was performed in a Q-Sonica cup horn sonication system using amplitude 70 with 30 s ON/OFF cycles for 10–15 min dependent on desired size and sonication efficiency of each sample. Samples were sonicated to a range of 100–500 base pairs. Sonicated chromatin was diluted in immunoprecipitation buffer. Two million cells were used for each immunoprecipitation (IP), with five consecutive IPs for each histone modification of interest. Ten percent of the initial sample volume per IP was set aside to serve as input control prior to addition of the appropriate antibody. Additional file 1: Table S1 provides the specifics for the antibodies used, with 2.5 μg of each antibody per IP. To isolate the antibody-bound fragments of interest, we used 50 μl of a 50/50 mix of Dynabeads TM Protein A (catalog # 10002D, lot # 00448844) and Protein G (catalog # 10003D, lot # 00486042) and an overnight incubation at 4 °C.

Beads were washed with RIPA buffer for five consecutive washes followed by a single wash with Tris-EDTA buffer. Complexes were eluted from the beads with 50 mM Tris, 10 mM EDTA, 1% (w/v) SDS, pH 8.0, and crosslinks were reversed. Concentration of resultant DNA was determined using Qubit according to manufacturer’s protocol. ChIP-seq libraries were prepared using DNA SMARTTM ChIP-Seq Kit. Sequencing was performed using Illumina HiSeq 2500 generating single end reads of 50 base pairs. Sequences were aligned to mouse genome assembly (mm9) using Bowtie2 v2.1.0 with default settings [30]. Using the Bedtools software suite for genome arithmetic [31], we determined the degree of enrichment of reads genome-wide. For data visualization, we used R software suite. To visualize enrichment patterns of histone modifications at promoters and enhancers, we used ngs.plot [32], using enhancer annotations from a previous report [33].

### Protein-protein interaction networks

Protein-protein interaction networks (PPIs) were constructed with the STRING database using all differentially expressed genes between male and female ES cells (STRING version 10.5 [34]). To improve the quality of the resulting network, the “minimum required score” option was set to 0.7 and “text mining resources” were ignored. Analysis and graphing of the networks were performed in the Gephi software (version 0.9.2) [35]. Functional modules were detected using an algorithm to partition the network into communities of densely connected nodes [36]. Gene ontology (GO) analysis was performed using the ClueGO plug-in from Cytoscape [37]. GO terms were summarized using the REVIGO website (http://revigo.irg.hr/revigo.jsp) [38]. Network topology analysis and selection of important genes were done as previously described [39, 40].

### ES cell differentiation

Two of the male and female ES cell lines from which ChIP-seq was performed were subjected to a standardized differentiation protocol that directs the stepwise differentiation of early embryonic cells into cardiac precursors [41], as assayed by marker gene analysis. Cells were cultured with leukemia inhibitory factor (LIF) on mouse embryonic fibroblasts (MEFs). Before differentiation, ES cells were dissociated, MEFs were eliminated as detailed above, and embryoid bodies were derived by hanging drop culture in medium without LIF. After 4 days, embryoid bodies were harvested and grown in medium containing Activin A, BMP4, and VEGF as monolayers until beating foci were observed. This optimized protocol yields > 75% cardiomyocytes [41]. At day 13 of initial LIF withdrawal, we picked beating foci from the plates and obtained RNA.

qPCR was performed to determine expression of pluripotency markers *Nanog* and *Oct4* and cardiomyocyte markers *Myh6* and *Tnnt2* on cDNA generated using SuperScript^TM^ II (Invitrogen) and relative expression was assessed using PowerUp SYBR Green Master Mix (Thermo Fisher) and normalized to *β*-actin on the Applied Biosystems StepOnePlus Real-Time PCR System. RNA-seq was performed as previously described [24].

### Meta-analysis of publicly available data

We leveraged existing expression datasets from across cardiac development in the mouse and stratified the data by sex when necessary. These collated data allowed us to determine whether there is dynamic sex-biased expression across cardiogenesis. Additional file 2: Table S2 details all the datasets examined herein.

Single cell data for 8.5, 9.5, and 10.5 days post coitum (dpc) embryonic [42] and neonatal mouse hearts [43] were downloaded and processed as follows: (1) if the fragments per kilobase of exon per million reads mapped (FPKM) was < 1, the gene was designated as not expressed; (2) genes with zero variance across all cells were removed. Cells were then sexed by determining the ratio of *Xist* to *Eif2s3y*, two oppositely biased genes, on a cell-by-cell basis. Cells with *Xist*/*Eif2s3y* ratios of at least 1.5 were considered as female and ratios below 1 were taken as male. *t* test analysis was performed on the samples from each stage, *p* values were used to calculate the false discovery rate (FDR), and genes with adjusted *p* value < 0.05 were selected as differentially expressed genes. Data for adult mouse hearts was already stratified by sex [44].

### Transcription factor binding analysis

To detect recognition motifs of candidate transcription factors (TFs) in genes enriched in male or female cells, we used the genome-wide position matrix scanner from the Computational Cancer Genomics website (https://ccg.epfl.ch/pwmtools/pwmscan.php) with the JASPAR core vertebrate motif library (version 2018). We searched for *Lef1* MA0768.1 and *Zeb1* MA0103.3 motifs with a *p* value cutoff of 0.00001 with the Contra v3 tool (http://bioit2.irc.ugent.be/contra/v3/#/step/1) and uploaded the results as custom tracks in the UCSC browser.

## Results

### Defining a gene network associated with sex-biased gene expression

There is a substantial number of differentially expressed genes in male (40, XY) and female (40, XX) mouse embryonic stem (ES) cells, including transcription factors (TFs), and epigenetic and remodeling enzymes (EREs) [22–24]. Yet both male and female ES cells are pluripotent and can contribute to normal development. Thus, while overall pluripotency networks govern both XX and XY ES cells, we hypothesized that differentially expressed genes can shift network architecture or constitute subnetworks with distinct gene-gene correlations.

To determine whether the genes differentially expressed in XX and XY ES cells constitute sex-specific co-expression networks and identify genes with higher connectivity in each sex, we used normalized RNA-sequencing (RNA-seq) data from six male and six female mouse ES cell lines to perform weighted gene co-expression network analysis (WGCNA) [24, 27, 45] (see “Methods” section). Weighted gene co-expression network analysis allows partitioning of genes into modules that correlate with biological function and identifies the genes that are most likely to be crucial in regulating that function. WGCNA has been successfully applied to dissect the role of hormonal and sex chromosome effects in sex-biased co-expression networks in adult tissues [46].

**Fig. 1:**
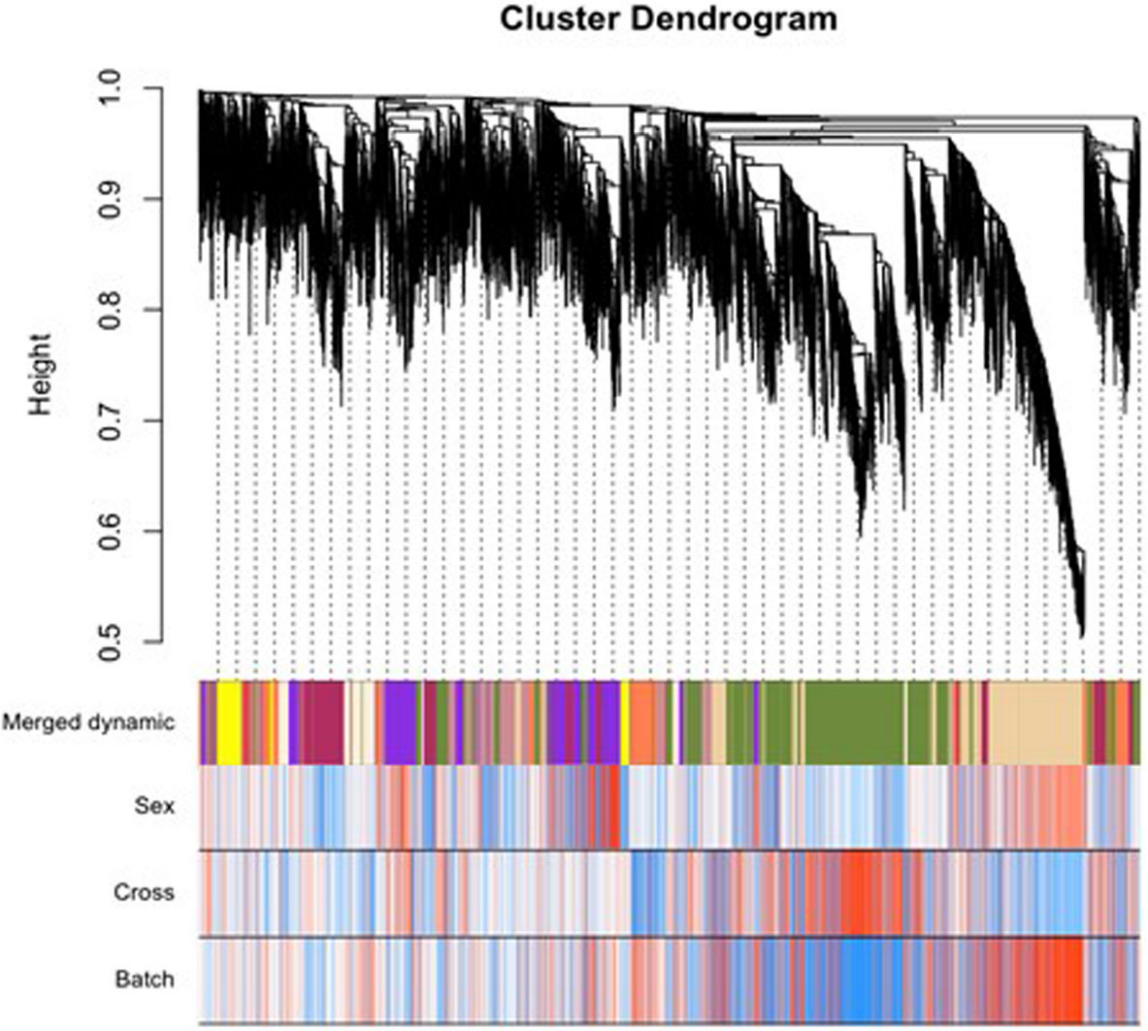
Weighted gene co-expression network analysis (WGCNA) for male and female ES cells. Expression modules were identified by weighted gene co-expression network analysis. Gene dendrograms display the co-expression modules identified by WGCNA from expression data from 6 male and 6 female ES cell lines and labeled by different colors. Dendrograms were generated by unsupervised hierarchical clustering of genes using topological overlap to identify co-expressed genes in modules. The significantly preserved modules are denoted by the striped colors in the bars below the dendrogram along the x-axis, referred to as the merged dynamic. The bars below the merged dynamic express correlation with sex, cross and RNA-seq batch. The y-axis shows the heights where the clusters merged

Figure 1 shows the clustering dendrogram of co-expressed genes resulting from the WGCNA with the lowest power value for scale-free topology, β, set at 10. To avoid bias by pre-selecting genes with differential expression levels in male and female ES cells, we based the clustering on all aligned transcripts. Genes with similar patterns of expression were grouped into modules by hierarchical average linkage clustering using topological overlap [26]. The initial dynamic tree cut was further merged, to generate a subset of 11 distinct co-expression modules.

**Fig. 2:**
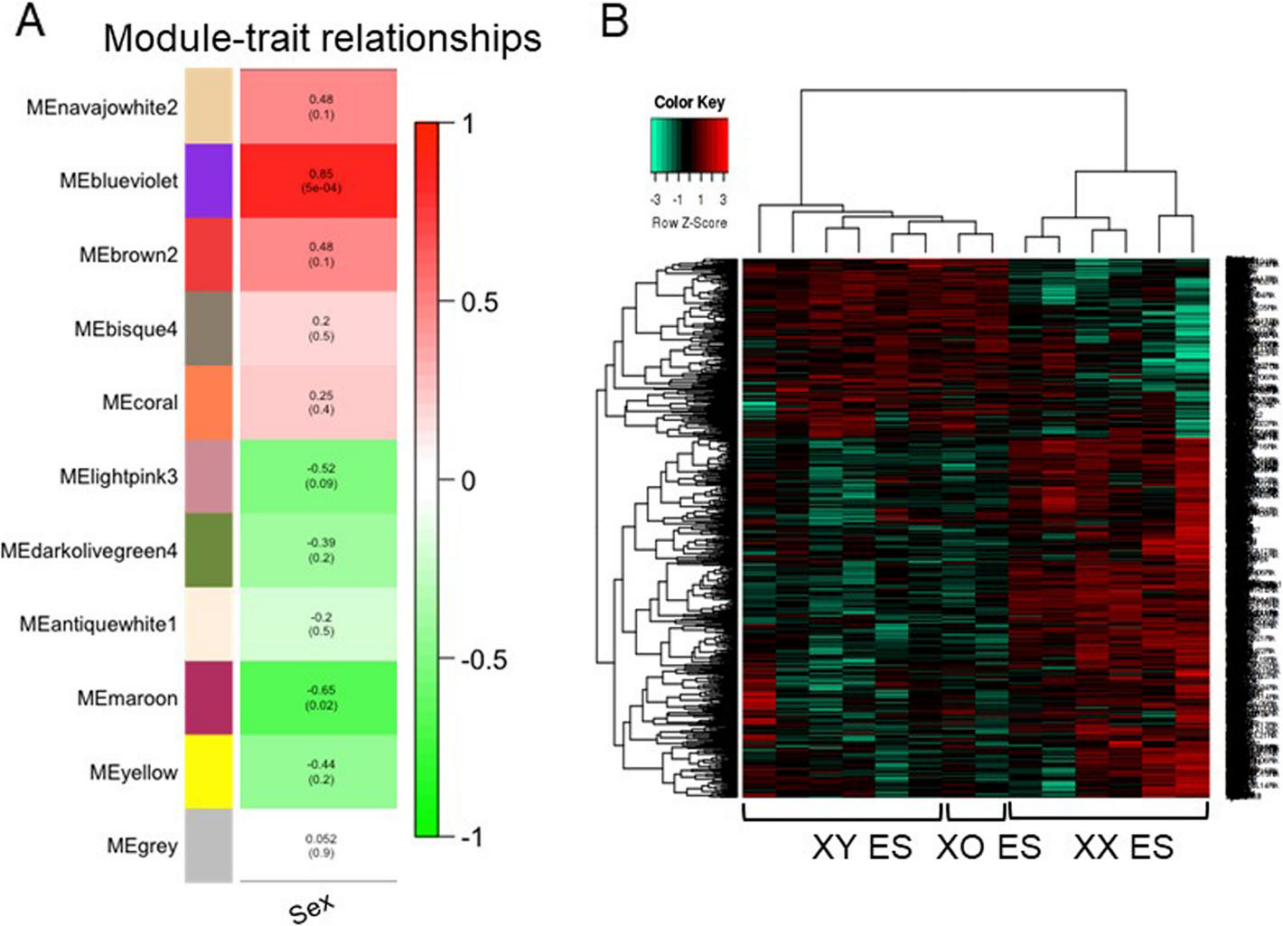
Relationships of consensus modules (module eigengenes) with sex. **a** Each row in the table corresponds to a consensus module identified by distinct colors along the left y-axis. Each module eigengene (ME) was evaluated in relationship to sex. Numbers in the table report the correlation of the corresponding ME with sex, with the *p* values shown in parentheses. The degree of correlation, positive and negative is provided by the colored scale on the right y-axis. **b** Clustering of the mouse ES cell lines based upon the module eigengene, blue/violet. Heatmap showing separation of the lines by sex chromosome complement (XY, male; XX, female; XO, X chromosome monosomic) when the 1624 genes, contained within the blue/violet module from WGCNA were evaluated

The first principal component of a given module is the module eigengene (ME), which represents the gene expression profile within that particular module. To understand the functional significance of the modules, we correlated the 11 MEs generated within the clustering dendrogram with traits of interest and isolated the most significant associations (Fig. 2a). According to the heatmap of module-trait correlations, sex showed a strong and independent association with a particular eigengene, the ME blue/violet (*r* = 0.85, *p* = 5e-04) and consisted of 1624 genes, including 84 TFs and 43 EREs (Additional file 3: Dataset S1).

To validate the gene cluster with a separate method, we produced a hierarchical clustering heatmap using the expression levels of the 1624 genes in the blue/violet module. Input of the genes contained within the blue/violet module into this separate pipeline did indeed show separation of the mouse ES cell lines by sex (Fig. 2b).

### Distinct upstream regulators are associated with sex-biased functional pathways

To identify regulatory pathways for the genes in the blue/violet module (Fig. 2), i.e., the module best correlated with sex, we performed ingenuity pathway analysis independently on the XX- and XY-enriched TFs and EREs (Additional file 4: Dataset S2). We found that the top pathway for XX-enriched TFs and EREs was “DNA methylation and transcriptional repression” (*p* = 7.81 e^−4^), with Max and Mycn as the top upstream regulatory molecules. Analysis of the XY-enriched TFs and EREs from the blue/violet module identified “Jak1 in Interferon Signaling” as the top pathway (*p* = 2 e^−3^). Top upstream regulators were predicted to be Irf9 and Npc1.

### Prdm14 motifs are enriched in promoters of sex-biased genes

We asked if the sex-biased clustering of the blue/violet genes was being driven by specific transcription factors and reflected sex-specific regulatory networks. To test this, we used HOMER to identify known transcription factor binding sites within the gene set in the blue/violet ME [28].

HOMER motif analysis yielded significantly enriched TF motifs in the promoters of genes in the blue/violet module eigengene (Table 1). The transcription factor TEAD (TEA/ATTS domain) was the top and most significantly enriched motif (*p* value 1e-15). TEAD proteins are pivotal transcription factors implicated in development as well as in cancer [47]. Leukemia inhibitory factor, present in the culture medium, activates the Yes-associated protein (YAP) and TEA domain TEAD2 transcription factor pathway, which contributes to mouse ES cell maintenance of pluripotency and self-renewal. The stem cell factors Nanog and Oct3/4 are targets of the TEAD-pathway [48]. These pluripotency factors had similar expression levels among all male and female ES cell lines tested, and TEAD was not differentially expressed at the RNA level. However, it was previously reported that Tead1 and Tead2 are male-biased at the protein level [23]. Thus, further investigation is required to determine if these factors contribute to the sex-specific effects or whether they appear with the HOMER analysis due to their contribution to pluripotency per se.

**Table 1:**
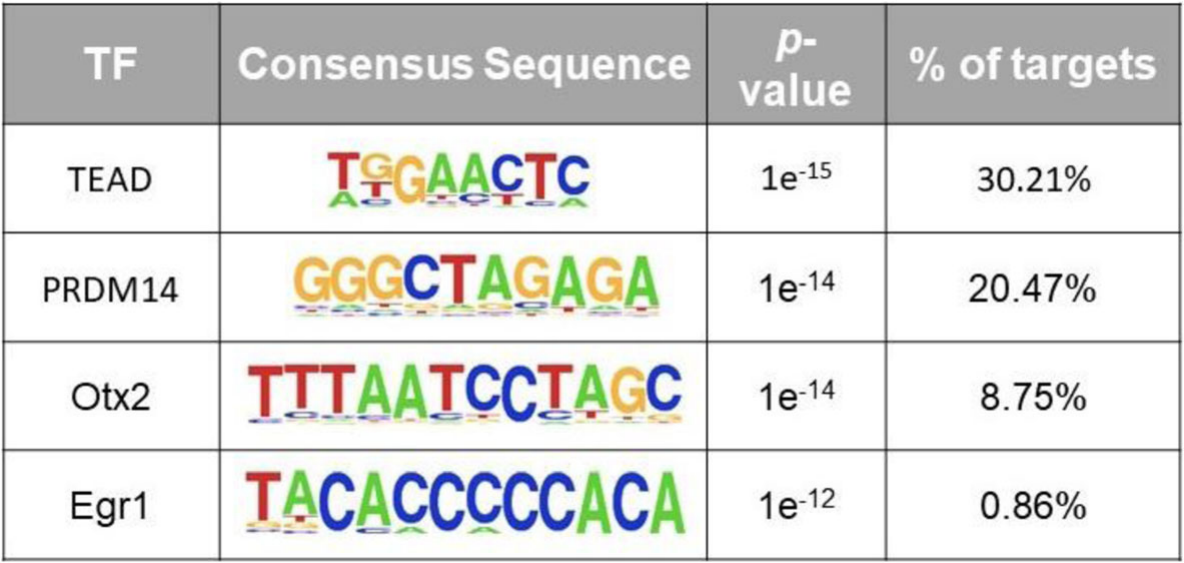
HOMER motif analysis of the promoters of genes in the blue/violet module eigengene

Genomic regions for − 5000 bp to + 1000 bp around the transcriptional start site were pulled for each of the 1624 genes contained within the blue/violet module eigengene and input into HOMER. Shown here are the four top hits and corresponding *p* values and percentage of coverage within target sequences

Interestingly, Prdm14 is a top hit and the second highest hit in HOMER (Table 1). *Prdm14* is expressed more highly in female (XX) than in male (XY) ES cells, a bias that occurs independently of whether the ES cells are cultured in LIF/serum or 2i [22, 24] and is also seen at the protein level [23]. Prdm14 is a bi-functional TF with a cardinal role in ES cell pluripotency and in the establishment of primordial germ cells. Prdm14 can either activate or repress gene expression, depending on its interacting partners [49]. Recruitment of polycomb repressive complex 2 (PRC2) by Prdm14 results in transcriptional repression, whereas cooperation with estrogen-related receptor β (Esrrβ) activates target gene expression. However, the mechanisms by which Prdm14 selectively partners with its alternative co-factors, resulting in gene activation or repression, are not understood. Nevertheless, Prdm14 is a strong candidate for regulating gene expression differentially in male and female ES cells and establishing sex-biased epigenetic marks.

### Prdm14 target genes encoding TFs have sex-biased expression

To identify downstream targets of Prdm14, we curated and compared publicly available expression profiles of ES cells depleted of Prdm14, focusing on TFs and EREs. Several studies have reported *Prdm14* knockout or knockdown in ES cells, with inconsistent results, likely due to varying culture conditions, strains, and karyotypes [50–52]. Therefore, we focused on a report with siRNA-mediated knockdown of *Prdm14* in wild-type female 129/Ola ES cells, with the caveat that culture conditions were 2i (versus LIF/serum in our lab) [50].

ES cells depleted of *Prdm14* have a more “male-like” expression pattern, with upregulation of *Foxi3*, *Sox11*, *Gata4*, *Dnmt3a*, and *Dnmt3l*, which are highly expressed in wild-type male ES cells. Genes that are downregulated in *Prdm14*-depleted female ES cells, such as *Mitf*, *Zeb1*, and *Prdm14* itself, are enriched in wild-type female ES cells. More than 10% of genes followed this pattern. This confirms that Prdm14 regulates a subset of genes, while also indicating that there are other factors involved in sex-biased expression.

### Male and female ES cells exhibit sex biases in chromatin modifications

**Fig. 3:**
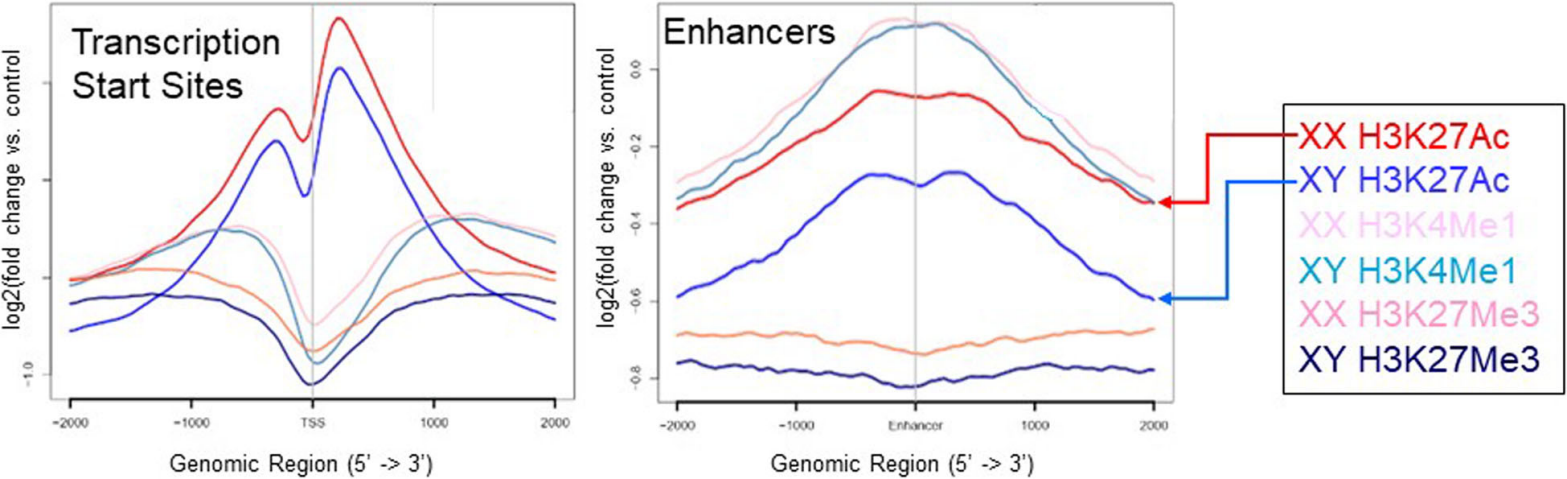
Sex-biased chromatin modifications at regulatory sequences in ES cells. ChIP-Seq results on two XX (red, light pink, dark pink lines) and XY (blue, teal, and dark blue lines) ES cell lines for H3K27Ac, H3K27Me3, and H3K4Me1, respectively, are shown. IgG served as a control. NGS-plot was used to evaluate the enrichment of the histone modifications at transcriptional start sites and known enhancers. The plots depict the average profile of histone modifications at regions of interest, providing a quantitative view of the patterns for each ES cell line

To determine if the differential transcriptomes between XX and XY ES cells are reflected in the chromatin structure, we performed chromatin immunoprecipitation and sequencing (ChIP-seq) on six early passage independent ES cell lines of each sex, i.e., the same cell lines for which we had reported sex-biased expression [24]. Antibodies against histone modifications H3K4Me1, H3K27Me3, and H3K27Ac were used to precipitate chromatin substrates with our standard protocol. The presence of H3K27Ac, indicating active chromatin, showed a significant difference between XX and XY ES cells at known enhancer regions (Fig. 3)*.* This suggests that the main biases between XX and XY ES cells are established by TFs and EREs that bind and modify enhancer sequences.

To determine whether there was concordance between Prdm14 binding, gene expression biases, and differential chromatin modifications, we integrated available Prdm14 ChIP-seq data in ES cells [50] with our sex-specific chromatin studies for genes that respond to Prdm14 according to the knockdown studies.

**Fig. 4:**
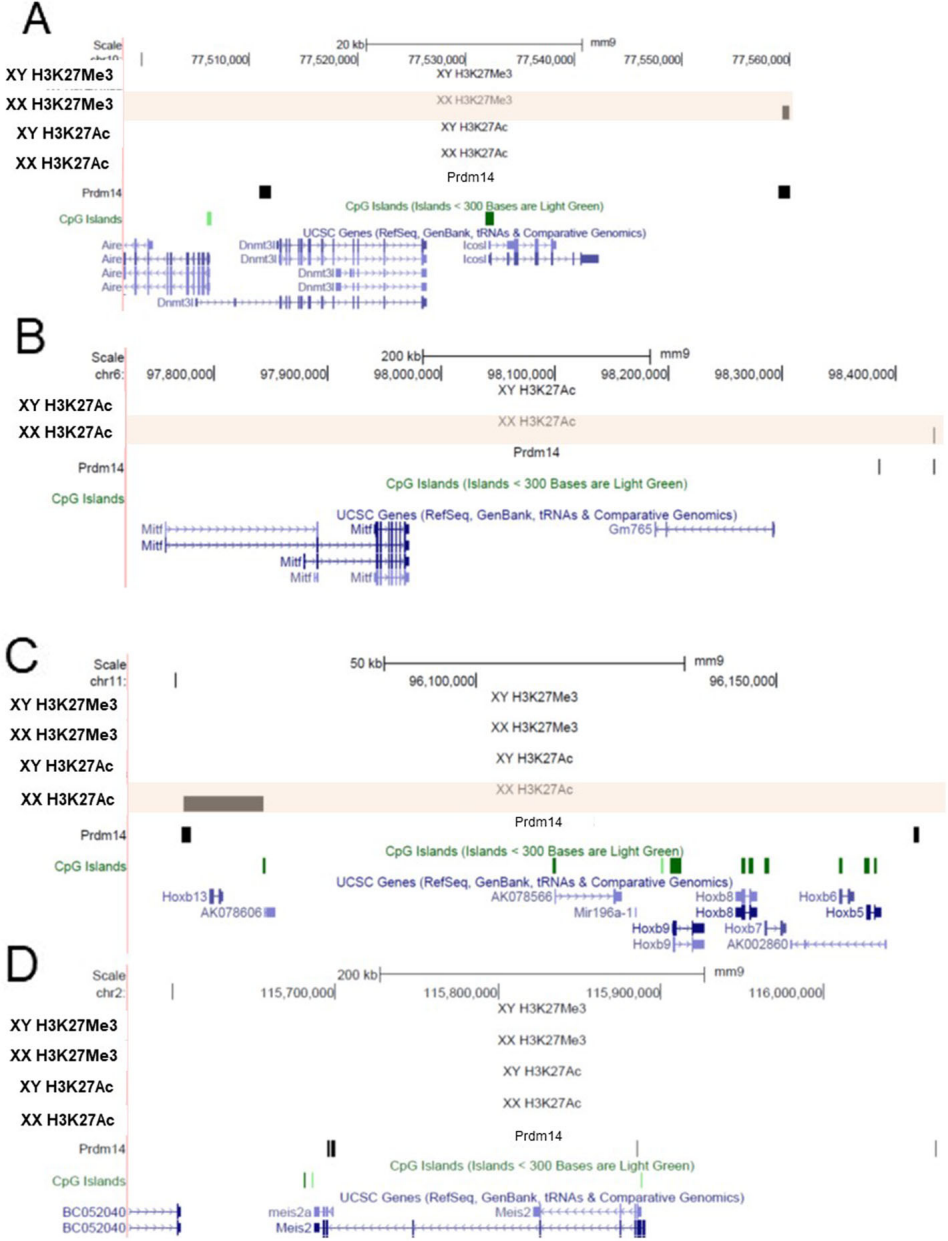
Differential H3K27Ac and H3K27Me3 enrichment in male and female ES cells. UCSC browser screen shots are shown with tracks denoting chromatin status designated as XY or XX. Black bars indicate presence of an enriched mark or Prdm14 binding in the corresponding track. Prdm14 occupancy track in ES cells was obtained from Ma et al. Browser shots for **a** *Dnmt3l*, **b** *Mitf*, **c** *Hoxb9*, and **d** *Meis2*

Our analysis identified three groups of differentially expressed genes: (1) genes that exhibited sex-biased chromatin modifications and Prdm14 binding, (2) genes with Prdm14 occupancy and no sex-specific histone modifications, and (3) genes with neither detectable sex-biased chromatin modification nor Prdm14 occupancy. For example, *Dnmt3l*, more highly expressed in male ES cells, has a *Prdm14* binding site 40 kb downstream of the transcription start site, which is enriched in H3K27Me3, a repressive mark, in female ES cells (Fig. 4). One of the *Prdm14* binding sites downstream of *Mitf*, more highly expressed in XX ES cells, has enrichment of H3K27Ac in those cells. *Hoxb9* shows a similar pattern, with a *Prdm14* binding site enriched in H3K27Ac in female ES cells which have higher expression. On the other hand, there are several *Prdm14* binding sites upstream and in the promoter of *Meis2*, but we did not detect differential histone modifications in male and female ES cells, although it is more highly expressed in female cells. Genes such as *Sohlh2* do not have apparent *Prdm14* binding in their vicinity, indicating that they are regulated by other, as yet unknown TFs.

### Protein-protein interaction network analysis of ES cell transcriptomes reveals overlap with Prdm14 target genes

**Fig. 5:**
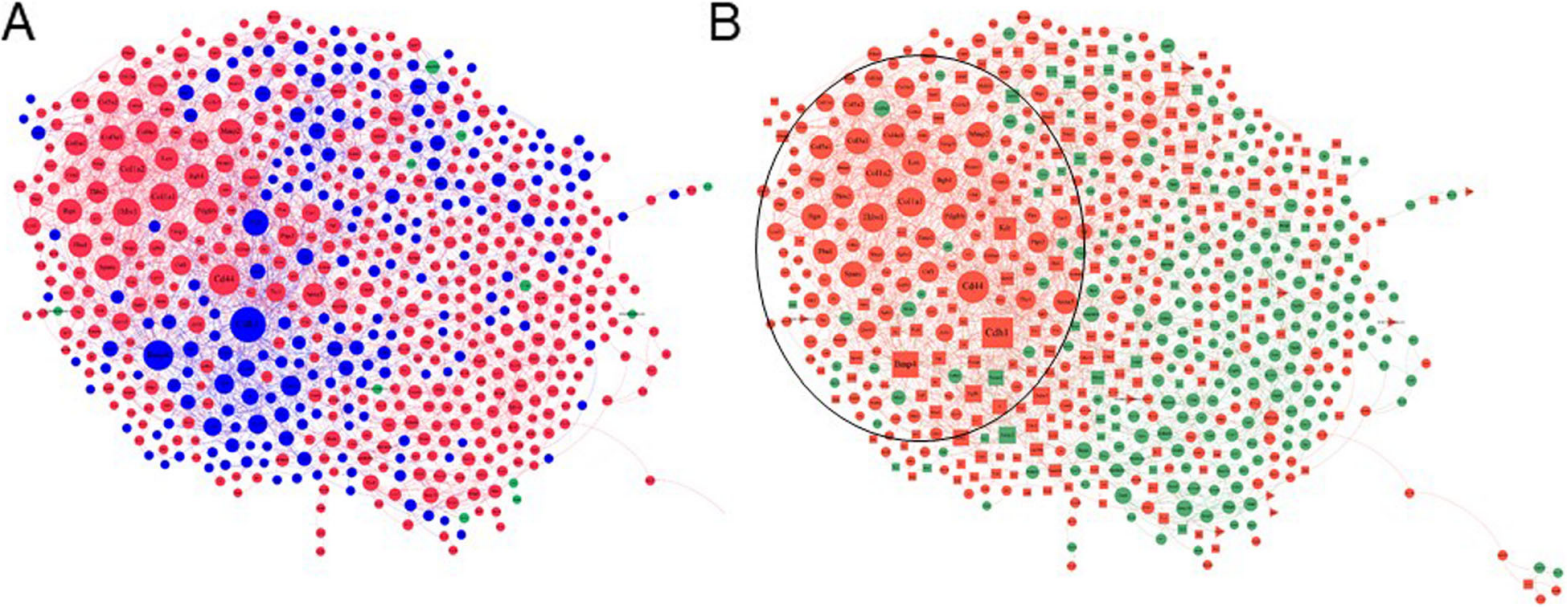
Protein-protein interaction networks. **a** PPIs were constructed from differentially expressed genes from male and female ES cells. The networks includes sex-biased modules highlighted by red (female-enriched) and blue (male-enriched) nodes. **b** PPI network compared to genes in the blue/violet module from the WGCNA analysis. The most important module (based on topological analysis) is encircled. The common genes are green, unique genes are orange; squares represent male-biased and circles female-biased genes

Data from the differentially expressed genes in male and female ES cells were used to construct a protein-protein interaction (PPI) network (Fig. 5a). Overlaying the information from the sex differences in gene expression shows that there are sex-biased modules within the global interaction network. We compared the genes from the blue/violet module eigengene from the WGCNA to the nodes of the PPI network. Two hundred twenty-five genes were shared between them (green nodes in Fig. 5b). Analysis of the network revealed six modules (Additional file 6: Figure S1), one of which contained the most important nodes based on topological analysis (degree, betweenness, and closeness centrality metrics in Additional file 5: Dataset 3). GO analysis of this module showed “blood vessel morphogenesis” and “Bmp signaling” as top terms (Additional file 7: Dataset S4).

Prdm14 was contained in the module with the most important nodes and showed connections to Dazl, Tcl1, Wnt3, Cdx2, Dnmt3b, Prdm6, Bmp4, Lin28a, Lefty2, T, and Gata4. Strikingly, many of the nodes in this PPI module, such as Zeb1, Lefty1, Gata4, Dusp6, and Sox11, are direct Prdm14 transcriptional targets in ES cells.

### Male and female cardiac precursors also show sex biases in gene expression

Thus far, we showed that there are sex-specific expression and protein-protein interaction networks in ES cells. Upon differentiation of female ES cells, one of the two X chromosomes is inactivated, a massive epigenetic event that equalizes most of the X-linked genes between males and females. This transition mirrors the in vivo process of blastocyst implantation, during which female embryos undergo X chromosome inactivation (XCI).

**Fig. 6:**
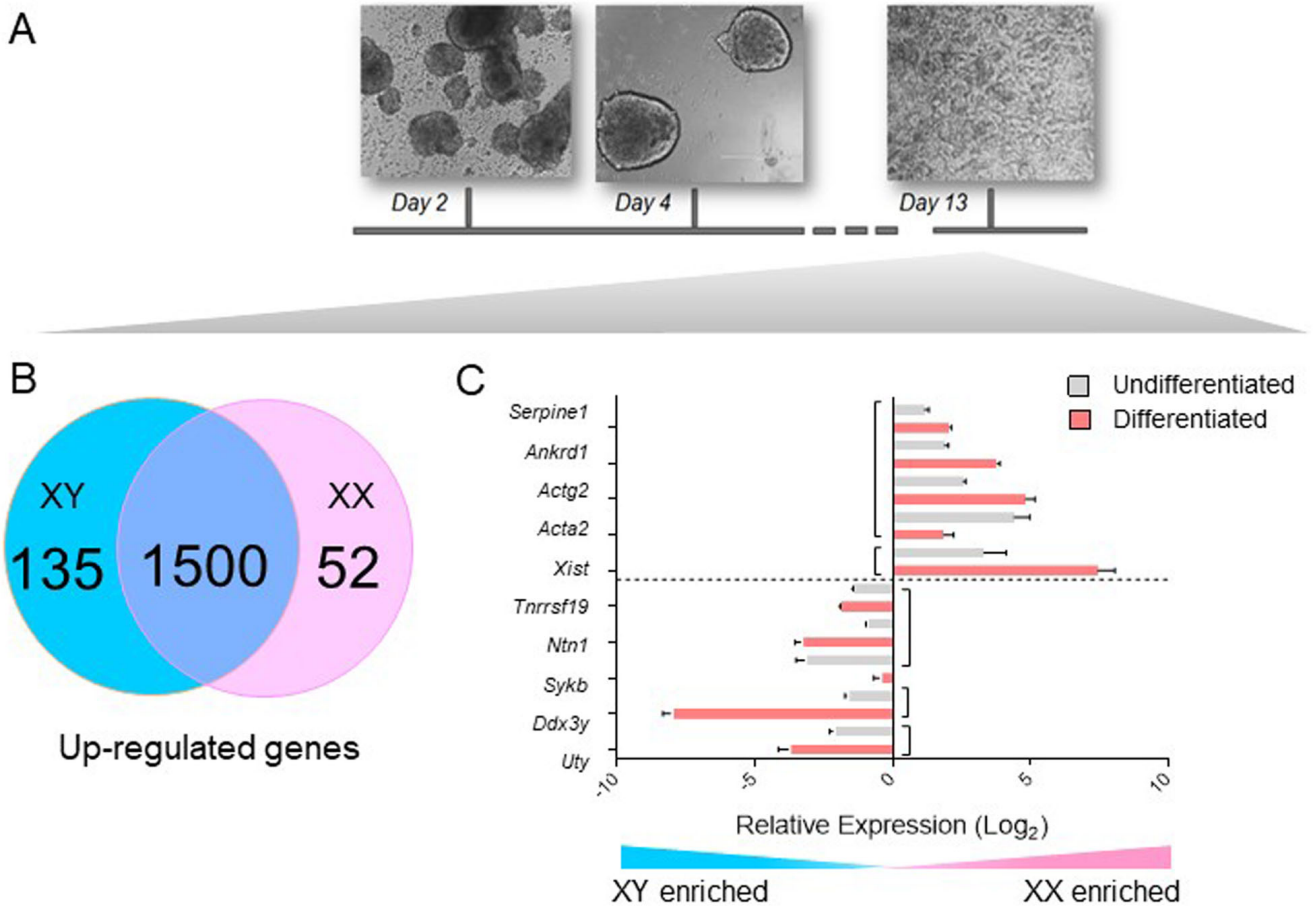
Differentiation of male and female ES cells into cardiac precursors. Top, images resulting from differentiation of ES cell lines according to standard protocol, with beating cardiac precursor cells at day 13 of LIF withdrawal. Below left, comparison of up-regulated genes between XX and XY ES cell lines indicating common and sex-specifically expressed RNAs (*q* < 0.01). Below right, expression of a subset of sex-biased genes expressed before and after differentiation of ES cells as analyzed by qRT-PCR in undifferentiated ES cells (gray) and derived cardiac precursors (coral). Error bars represent S.E.M. of duplicate experiments with three replicates each

To determine whether some sex-biased expression differences were perpetuated after XCI and during the beginning stages of lineage determination, we subjected two male and female ES cell lines to an optimized differentiation protocol to generate cardiac precursors and performed RNA-seq (Fig. 6). At day 13 after LIF withdrawal, ES cells have differentiated to cardiac precursors corresponding to 8.5–9.5 days post coitum (dpc) cardiac progenitors in vivo. RT-PCR confirmed that stem cell markers such as *Nanog* and *Oct4* were downregulated, whereas markers of cardiac differentiation, such as *Tnnt2* and *Myh6*, were upregulated in both sexes, as previously reported (Additional file 8: Figure S2) [10, 53].

We compared transcriptomes between differentiated male and female cell lines and found 157 genes that were differentially expressed at a FDR < 0.01 (Additional file 9: Dataset S5). The *Xist* non-coding RNA, which is involved in X chromosome inactivation, was more highly expressed in female cells, as expected. The male cells showed higher expression of 2 Y chromosome-linked genes, *Ddx3y* and *Uty* (*Kdm6c*). Interestingly, four TFs were more highly expressed in male cells, *Ferd3l*, *Pou3f3*, *Six6*, and *St18*. *Ferd3l* and *Pou3f3* have nearby *Prdm14* binding sites in undifferentiated ES cells, although we did not detect differential histone modifications in their vicinity (Additional file 10: Figure S3). Overall, this data shows that although the number of genes exhibiting sex differences diminishes during lineage determination, some biases persist. ChIP-seq data for cardiac precursors derived from male and female ES cells are needed to determine which epigenetic differences also persist after differentiation.

### Sex biases in cardiac expression exist in early cardiac developmental stages in vivo

To elucidate how sex biases in gene expression vary during cardiac development, we collated and analyzed single cell transcriptional profiles from mouse embryonic hearts at 8.5, 9.5, and 10.5 dpc [42] and post-natal day 1 (p1) (Additional file 2: Table S2) [43]. Single cell data was downloaded and sexed (Additional file 11: Dataset S6). We found that there were hundreds of sex-biased genes at every stage. Some of these were stage-specific and some were common to two or more time points. For example, *Lef1* was more highly expressed in male than female ES cells, and the same was true for 8.5 dpc and p1 hearts. *Tbx20* was also enriched in male ES cells, cardiac precursors, and in 10.5 dpc and p1 hearts.

The majority of genes with sex-biased expression were male-biased at every stage. The number of female-enriched genes peaked dramatically at 9.5 dpc and decreased thereafter. At 8.5 dpc, only three X-linked genes, including *Xist*, were female-biased, whereas 19 X-linked genes were male-biased. At 10.5, eight X-linked genes were more highly expressed in females, including *Xist*, *Tsix*, and three genes that had not been characterized as escapees. More than 30 X-linked genes showed male-biased expression, indicating that some genes are not dosage compensated by X chromosome inactivation, at least at this stage in this tissue.

**Fig. 7:**
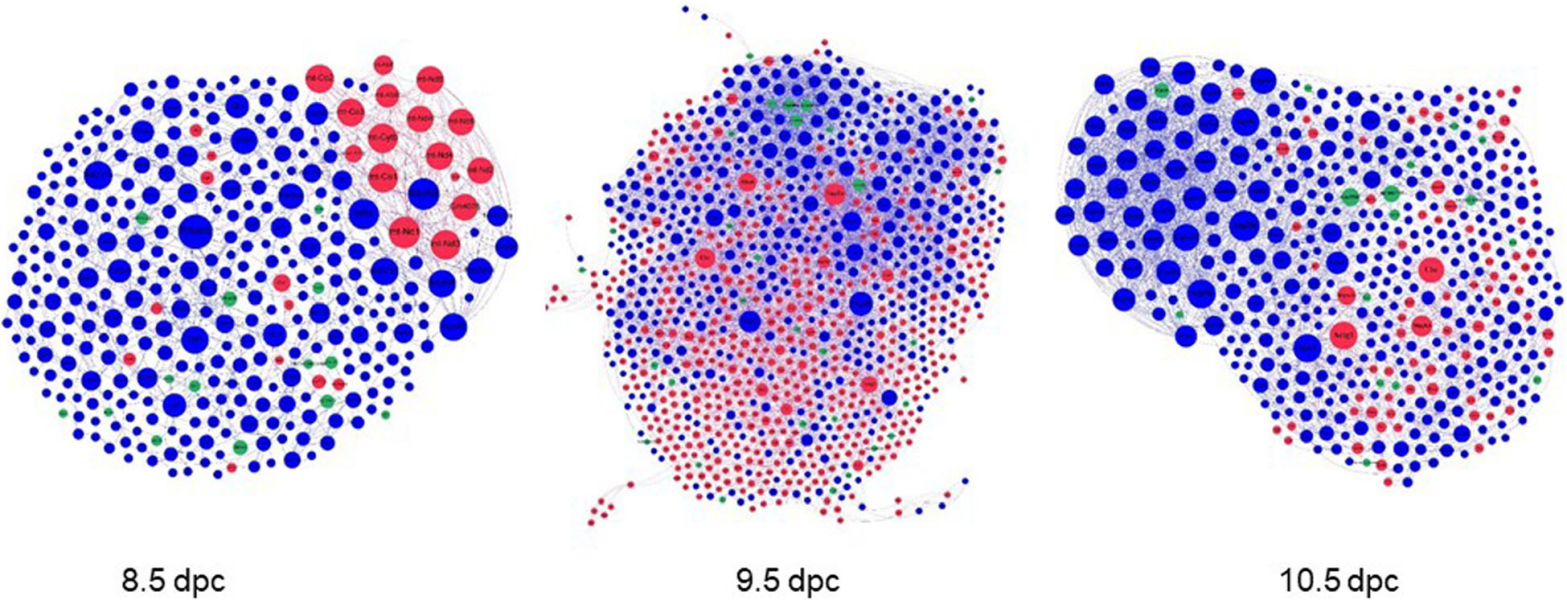
Protein-protein interaction networks in early cardiac development. PPIs were constructed from differentially expressed genes in 8.5, 9.5, and 10.5 dpc hearts as assayed by single-cell RNA-seq. Networks include sex-biased modules highlighted by red (female-enriched) and blue (male-enriched) nodes (based on data from Li, G. et al.)

Protein-protein interaction networks were constructed with the sex-stratified expression data from 8.5, 9.5, and 10.5 dpc hearts (Fig. 7, Additional file 12: Figure S4, Additional file 5: Dataset S3). Sex biases in specific modules varied across the developmental stages, suggesting a highly dynamic but constant pattern of sexual dimorphism at the molecular level.

### Adult male and female hearts have sex-specific pathways

To explore whether there are expression differences in male and female adult C57BL/6 mouse hearts, we inspected recently published transcriptomic data across 17 tissues, stratified by sex [44]. Strikingly, 908 and 148 genes exhibited expression biases in adult male and female hearts, respectively, again showing that male-biased genes are more numerous. Interestingly, 38 X-linked genes were male-biased, suggesting male-specific regulation of these genes.

We investigated whether TFs that were sex-biased in adult hearts showed Prdm14 binding in ES cells. We found that *Nkx2.5*, *Lef1*, *Id2*, *Ikzf3*, and *Srebf2* had Prdm14 occupancy in or near their promoter regions (Additional file 13: Figure S5), suggesting that their sex differences could have been established in early development. Differential histone modifications, however, were not apparent in ES cells in these regions.

We used Ingenuity Pathway Analysis to identify enrichment of biological network components in the sex-specific gene signatures in the adult heart. The top canonical pathways differed between male and female cardiac cells (Additional file 14: Datasets S7 and S8). Cardiovascular disease was the top disease association and cardiovascular system development and function was one of the top networks in importance for females, but surprisingly, cancer was the top disease association as well as the top network for males. Regulatory component analysis predicted distinct upstream regulatory factors for the male and female expression patterns. For example, Tp53, Nr3c2, and Tbx5 were among the top transcriptional regulators for female cells, whereas Ncor1 and Smad3 were identified for male cells.

### Conserved sex-biased expression between mouse and human hearts

We compared sex-biased genes in adult heart ventricles between mouse and human. Differentially expressed genes between male and female human hearts were obtained from DeMeo et al., in which expression from the GTEx portal was stratified by sex [4]. There are 70 and 328 genes that are enriched in females and males, respectively, in both mouse and human (Additional file 15: Dataset 9). Among these are TFs Bhlhe40, Tcf15, Npas3, and Mafa, which are enriched in female hearts. Males show higher levels of Ehf, Etv1, Foxk1, Ikzf2, Meis2, and Tbx20, among others and of EREs Hat1, Cdyl, and Rad54l2.

### There is sex-biased expression at specific developmental time points for important cardiac regulators

**Fig. 8:**
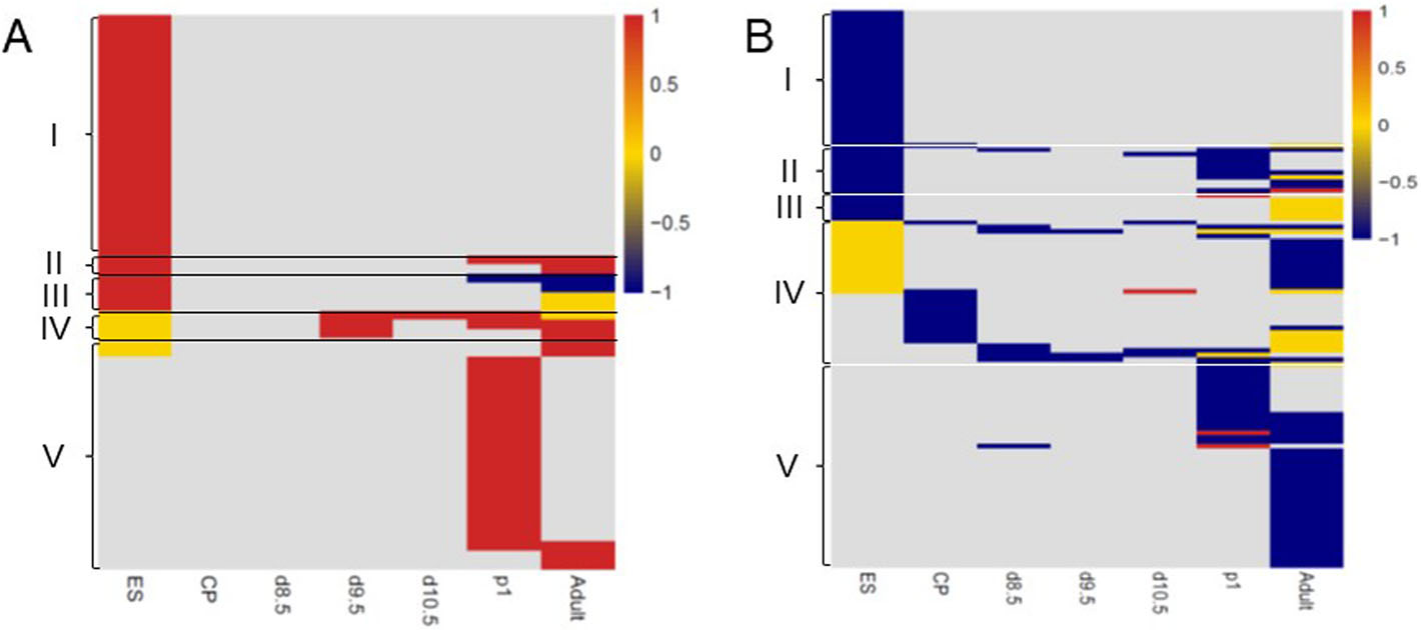
Expression of sex-biased transcription and epigenetic factors throughout development. Schematic heatmap representation of Tables 2 (**a**) and 3 (**b**) indicating expression and sex biases of transcription and epigenetic factors at each time point. Data was compiled from **a** female and **b** male ES cells, derived cardiac precursors (CP), hearts from 8.5, 9.5, and 10.5 days post coitum (dpc) embryos, neonates (p1), and adult mice (Ad). Each row is a specific transcription or epigenetic factor, with a total of 60 for females and 61 for males; the color denotes expression detected and enrichment in XX (red), XY (blue), or not biased (yellow). Group I: biased in ES cells, not expressed thereafter; groups II, III: biased in ES cells and same (II) or different (III) bias at other stages; group IV: biased after implantation but before gonadogenesis; group V: biased only after gonadogenesis

To query temporal changes in sex-biased expression profiles, we compared differential expression from ES cells (our data), embryonic and neonatal hearts, and adult cardiac myocytes [44]. Figure 8 and Tables 2 and 3 show sex-biased expression of TFs and EREs at each stage of heart development. Several different patterns can be visualized. Some genes encoding TFs and EREs are only expressed at one stage and others across several stages. For the latter group, there are subsets of genes that either maintain, acquire, lose, or even reverse their bias. A distinct group of genes, for example, *Carhsp1* (male-biased) and *Bhlhe40* (female-biased), display sex differences before gonad formation and the appearance of sex hormones. Our data also reveals sex disparities in expression that only become apparent in neonates and adults, suggesting that these respond, at least in part, to hormonal differences.

**Table 2:** Female-biased expression of transcription and epigenetic factors

Transcription factors and epigenetic remodelling enzymes	ES	CP	8.5	9.5	10.5	p1	Adult	Chr	Name
Hmgb3	XX							X	High mobility group box 3
Dmrtb1	XX							4	DMRT-like family B with proline-rich C-terminal, 1
Hoxb9	XX							11	Homeobox B9
Hoxc8	XX							15	Homeobox C8
Klf8	XX							X	Kruppel-like factor 8
Mitf	XX							6	Melanogenesis associated transcription factor
Prdm14	XX							1	PR domain containing 14
Rhox1	XX							X	Reproductive homeobox 1
Sohlh2	XX							3	Spermatogenesis and oogenesis specific basic helix-loop-helix 2
Spic	XX							13	Serine (or cysteine) peptidase inhibitor, clade B, member 6c
Tbx15	XX							3	T-box 15
Zeb1	XX							18	Zinc finger E-box binding homeobox 1
Zfp182	XX							X	Zinc finger protein 182
Zfp275	XX							X	Zinc finger protein 275
Zfp449	XX							X	Zinc finger protein 449
Zfp59	XX							7	Zinc finger protein 59
Zxdb	XX							X	Zinc finger, X-linked, duplicated B
Mecp2	XX							X	Methyl CpG binding protein 2
Nkap	XX							X	NFKB activating protein
Ogt	XX							X	O-linked N-acetylglucosamine (GlcNAc) transferase
Top2b	XX							14	Topoisomerase (DNA) II beta
Trim16	XX							11	Tripartite motif-containing 16
Zmym3	XX							X	Zinc finger, MYM-type 3
Apobec2	XX							17	Apolipoprotein B mRNA editing enzyme, catalytic polypeptide 2
Baz2b	XX							2	Bromodomain adjacent to zinc finger domain, 2B
Prrx1	XX	XX						1	Paired related homeobox 1
Kdm6a	XX					XX	XX	X	Lysine (K)-specific demethylase 6A
Lbh	XX						XX	17	Limb-bud and heart
Meis2	XX						XY	2	Meis homeobox 2
Zfp9	XX						XY	6	Zinc finger protein 9
Aff2	XX					XX	nb	X	AF4/FMR2 family, member 2
Atrx	XX					XY	nb	X	ATRX, chromatin remodeler
Arid1b	nb			XX	XX		nb	17	AT rich interactive domain 1B (SWI-like)
Bhlhe40	nb			XX			XX	6	Basic helix-loop-helix family, member e40
Zfp51	nb			XX			XX	17	Zinc finger protein 51
Klf15	nb						XX	6	Kruppel-like factor 15
Mafk	nb					XX	XX	5	v-Maf musculoaponeurotic fibrosarcoma oncogene family, protein K (avian)
Aff3	nb	XX		XX		XX	XY		AF4/FMR2 Family Member 3
Heyl						XX		4	Hairy/enhancer-of-split related with YRPW motif-like
Hopx						XX		5	HOP homeobox
Dlx1						XX		2	Distal-less homeobox 1
Rsl1						XX		13	Regulator of sex limited protein 1
Zbtb45						XX		7	Zinc finger and BTB domain containing 45
Zfp282						XX		6	Zinc finger protein 282
Zfp472						XX		17	Zinc finger protein 472
Zfp498						XX		5	Zinc finger and SCAN domain containing 25
Zfp758						XX		17	Zinc finger protein 758
Zkscan14						XX		5	Zinc finger with KRAB and SCAN domains 14
Zkscan6						XX		11	Zinc finger with KRAB and SCAN domains 6
Nat10						XX		2	N-acetyltransferase 10
Senp3						XX		11	SUMO/sentrin specific peptidase 3
Setmar						XX		6	SET domain without mariner transposase fusion
Sirt7						XX		11	sirtuin 7
Suv39h2						XX		2	suppressor of variegation 3-9 2
Bard1						XX		1	BRCA1 associated RING domain 1
Cbx2						XX		11	Chromobox 2
Cbx7						XX		15	Chromobox 7
Rcc1						XX		4	Regulator of chromosome condensation 1
Nkx2-5						XX	XX	17	NK2 homeobox 5
Irf4							XX	13	Interferon regulatory factor 4
Cecr2							XX	6	CECR2, histone acetyl-lysine reader

*ES* embryonic stem cells; *CP* cardiac precursors; 8.5, 9.5, 10.5 days post coitum (dpc) embryonic hearts (single cell); *p1* neonatal hearts; *Chr* chromosome; *empty cells* expression not detected; *nb* expressed but not biased

**Table 3:** Male-biased expression of transcription and epigenetic factors

Transcription factors and epigenetic and remodelling enzymes	ES	CP	8.5	9.5	10.5	p1	Adult	Chr	Name
Egr4	XY							6	Early growth response 4
Cdx1	XY							18	Caudal type homeobox 1
E2f7	XY							10	E2F transcription factor 7
Eomes	XY							9	Eomesodermin
Evx1	XY							6	Even-skipped homeobox 1
Foxi3	XY							6	Forkhead box I3
Foxp4	XY							17	Forkhead box P4
Insm1	XY							2	Insulinoma-associated 1
Lin28a	XY							4	Lin-28 homolog A (C. elegans)
Mesp1	XY							7	Mesoderm posterior 1
Mixl1	XY							1	Mix1 homeobox-like 1 (Xenopus laevis)
Nr6a1	XY							2	Nuclear receptor subfamily 5, group A, member 1
Pou2f3	XY							9	POU domain, class 2, transcription factor 3
Sox11	XY							12	SRY (sex determining region Y)-box 11
Sp5	XY							9	Per-hexamer repeat gene 4
Sp8	XY							12	Trans-acting transcription factor 8
T	XY							17	Brachyury, T-box transcription factor T
Tcf7	XY							11	Transcription factor 7, T cell specific
Wiz	XY							17	Widely-interspaced zinc finger motifs
Ybx2	XY							11	Y box protein 2
Zglp1	XY							9	Zinc finger, GATA-like protein 1
Dnmt3b	XY							2	DNA methyltransferase 3B
Dnmt3l	XY							10	DNA (cytosine-5-)-methyltransferase 3-like
Phc1	XY							6	Polyhomeotic 1
Plac8	XY							5	Placenta-specific 8
Tdrd5	XY							1	Tudor domain containing 5
Bahcc1	XY							11	BAH domain and coiled-coil containing 1
Kdm6c	XY	XY	XY	XY	XY		XY	Y	Ubiq.transcribed tetratricopeptide repeat gene, Y chr
Bcl6b	XY	XY					nb	11	B cell CLL/lymphoma 6, member B
Irf8	XY	XY					nb	8	Interferon regulatory factor 8
Arid1a	XY	XY						4	AT rich interactive domain 1A (SWI-like)
Lef1	XY		XY			XY	XY	3	Lymphoid enhancer binding factor 1
Tbx20	XY	XY			XY	XY		9	T-box 20
Smarcd1	XY					XY		15	SWI/SNF related, matrix assoc., actin-dep. reg. of chromatin d1
Zbtb7a	XY					XY		10	Zinc finger and BTB domain containing 7a
Hif3a	XY	XY				XY		7	hypoxia inducible factor 3, alpha subunit
Nfkb2	XY					XY	XY	19	nuclear factor of κ light polypeptide enhancer B cells 2, p49/p100
Pbx2	XY					XY	nb	17	Pre B cell leukemia homeobox 2
Prdm6	XY						XY	18	PR domain containing 6
Id2	XY						XY	12	Inhibitor of DNA binding 2
Dot1l	XY					XY	XX	10	DOT1-like, histone H3 methyltransferase (S. cerevisiae)
Zfp296	XY					XX		7	Zinc finger protein 296
Gata4	XY						nb	14	GATA binding protein 4
Mycn	XY						nb	12	v-myc avian myelocytomatosis related og, neuroblastoma derived
Hdac5	XY						nb	11	Histone deacetylase 5
Nfxl1	XY						nb	5	Nuclear transcription factor, X-box binding-like 1
Pax6	nb	XY						2	Paired box 6
Brd4	nb	XY					nb	17	Bromodomain containing 4
Carhsp1	nb		XY			XY	XY	16	Calcium regulated heat stable protein 1
Csde1	nb		XY	XY		nb	nb	3	Cold shock domain containing E1, RNA binding
Mef2d	nb					XY		3	Myocyte enhancer factor 2D
Atf5	nb						XY	7	activating transcription factor 5
Bach2	nb						XY	4	BTB and CNC homology, basic leucine zipper transcription factor 2
Cdc5l	nb						XY	17	Cell division cycle 5-like (S. pombe)
Crebzf	nb						XY	7	CREB/ATF bZIP transcription factor
Gm13139	nb						XY	4	Zinc finger protein 991
Hmgb1	nb						XY	2	Predicted gene, 21596
Hmgb2	nb						XY	8	High mobility group box 2
Klf10	nb						XY	15	Kruppel-like factor 10
Nfic	nb						XY	10	Nuclear factor I/C
Nfkb1	nb						XY	3	Nuclear factor of κ light polypeptide enhancer in B cells 1, p105
Pias2	nb						XY	18	Protein inhibitor of activated STAT 2

*ES* embryonic stem cells; *CP* cardiac precursors; 8.5, 9.5, 10.5 days post coitum (dpc) embryonic hearts (single cell); *p1* neonatal hearts; *Chr* chromosome; *empty cells* expression not detected; *nb* expressed but not biased

Thirty-six genes have conserved sex-biased expression in ES cells and adult hearts. Of those, six genes are more highly expressed in females at both stages, four of which are X-linked. Interestingly, only one of the X-linked genes has been previously described as escaping X chromosome inactivation (XCI) (*Kdm6a*) [54]. Thirty genes are male-biased in both ES cells and adult cardiomyocytes, including the three transcription factors *Nfkb2*, *Lef1*, *Id2*, and the epigenetic enzymes *Uty* and *Prdm6.*

Some genes expressed at early stages are still expressed in neonates or adults but lose their sex differences or even exhibit reversals in sex biases. In females, the X-linked *Aff2* and *Atrx* lose their bias, which likely reflects dosage compensation after X chromosome inactivation in female cells. However, *Meis2* and *Zfp9* switch to male-biased expression in adults (Table 2). Seven male-biased genes, including *Irf8*, *Pbx2*, *Gata4*, and *Hdac5*, which exhibit higher expression in male ES cells, become equally expressed in adult hearts of both sexes. *Dot1l* and *Zfp296* reverse their bias and are more highly expressed in females at later stages (Table 3).

We also find several genes that are not expressed differentially in male and female ES cells and later acquire a sex bias. These are good candidates for genes regulated by hormonal factors, although *Esr1*, the only estrogen receptor expressed in the heart, is not differentially expressed between males and females. RNA encoding the androgen receptor is also not sex-biased in the adult heart, suggesting that hormonal regulation depends on other co-factors and/or differential chromatin environment of the target genes.

**Fig. 9:**
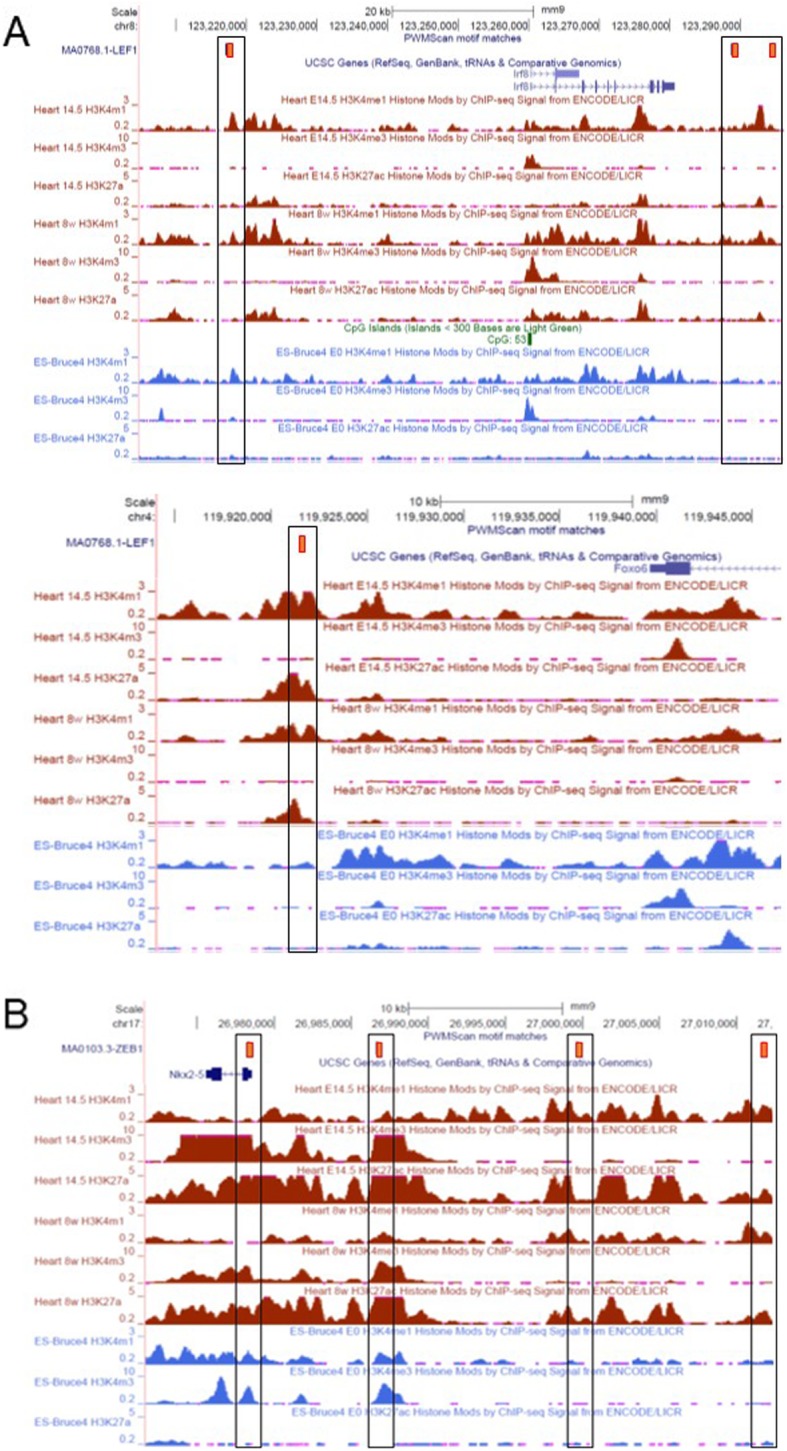
UCSC browser screenshots of genes regulated by sex-biased transcription factors (TFs). Custom tracks show TF binding sites for (**a**) Lef1 (male-biased) and (**b**) Zeb1 (female-biased) for genes that share the same bias with the TFs, with the binding sites denoted as orange bars. Also shown are the histone modification profiles for ES cells and 14.5 dpc and adult hearts, highlighting active histone marks coinciding with TF binding sites

To further explore the role TFs expressed in early development have at later stages, we identified binding sites for Lef1 and Zeb1 within the regulatory regions of genes differentially expressed between male and female cardiomyocytes. *Lef1* is enriched in male ES cells, 8.5 dpc embryonic hearts, and in neonatal and adult hearts. Genes that harbored *Lef1* binding motifs included other TFs that are also male-biased in ES cells, such as *Mixl1*, *Mesp1*, *Irf8*, and *Tbx20*, but also genes that are only later expressed in the adult heart, such as *Gata5* and *Foxo6*, which are also male-enriched (Fig. 9a). *Zeb1* is enriched in female ES cells and not detected at later stages, but its cognate motifs are present in genes that are female-biased in adult heart, such as *Cecr2* and *Nkx2-5* (Fig. 9b). These results suggest that TFs expressed in early development may determine sex-biased gene expression at later stages.

## Discussion

This study challenges the expectation that sex biases in gene regulation are non-existent during early mammalian development. Although sex determination has been traditionally associated with processes leading to distinct reproductive systems in males and females, we show that sex biases appear soon after fertilization and may have sex-specific repercussions during organogenesis, some of which persist in adults. In the absence of time-series experiments from pre-implantation embryos throughout lineage determination and organogenesis, we capitalized on our own data and a series of previously published RNA-seq datasets.

### Gene co-expression network analysis identifies Prdm14 as a key determinant of sex-biased gene expression in ES cells

Previous reports have identified thousands of genes differentially expressed in male and female ES cells and pre-implantation embryos in rodents, bovine, primates, and humans [21–24, 54–59]. In this work, we asked whether sexual dimorphism is detectable at the molecular level of gene expression and enriched in protein-protein interaction networks in early development. Both WGCNA and PPI networks revealed that important modules associated with sex are enriched in genes with *Prdm14* cognate binding sites and are *Prdm14* target genes.

*Prdm14* is important for pluripotency in ES cells [49, 52] and is a key regulator of primordial germ cell specification [60, 61]. In contrast to other PRDM family members, Prdm14 does not exhibit histone methyltransferase, but has been shown to partner with enzymes that catalyze post-translational modification of histones [49]. In fact, as seen in our ChIP-seq data, male and female ES cells have differential chromatin modifications, some of which are associated to *Prdm14* occupancy at regulatory sequences. In addition, Prdm14 binding is found in promoters or neighboring regions of genes that are not expressed in ES cells. Thus, epigenetic marks established in pre-implantation stages can potentially result in sex-biased gene expression later in development.

*Prdm14* expression is downregulated after differentiation of male and female ES cells and after implantation in vivo. However, female ES cells are developmentally delayed relative to male cells due to the process of X chromosome inactivation (XCI) [56]. Consequently, they are exposed to higher *Prdm14* levels for a longer period, which could lead to the establishment of female-specific epigenetic marks. In fact, we previously reported that a *Prdm14*-responsive enhancer exhibited higher activity in female ES cells, strongly suggesting that *Prdm14* target gene levels are dosage-sensitive [24]. In addition, it is possible that a subset of genes regulated by *Prdm14* is distinct in male and female ES cells. This is also true of any dose-dependent TF or ERE with sex-biased expression. Therefore, future ChIP-seq studies for TFs and chromatin modifications performed in a sex-stratified manner should allow us to distinguish between these possibilities.

Our studies also show that there are factors in addition to *Prdm14* that regulate sex-biased gene expression. For example, X-linked genes that are expressed from the two active X chromosomes, such as *Atrx*, *Kdm6a*, and *Klf8*, are strong candidates for involvement in sex-biased expression. However, autosomal factors, such as *Lef1* and *Zeb1*, could be involved as well. In theory, TFs that are not sex-biased could also be important for differential gene expression and according to our network analysis (WGCNA), there are a host of other TFs and EREs that are significantly correlated with sex such as Arid3b, Smad4, Jarid2, and Kdm8. For regulatory factors that are not sex-biased per se, their cognate sites could present different accessibility in male and female cells or there could be differential availability of their co-factors.

### Differentiated ES cells exhibit sex-biased gene expression

Differentiation of male and female ES cell lines into cardiac precursors drastically changed the transcriptional profile of the cells, but we still detected sex-biased expression. Most X-linked genes were expressed equally between male and female cells due to the process of XCI, but unexpectedly, four were more highly expressed in male cells, suggesting that there is male-specific regulation of some X-linked genes. Some of the sex differences in gene expression could represent the slight developmental delay of the female cells. Yet some expression differences observed in ES cells persist in the adult heart, suggesting that these are independent of developmental stage and are integrating *bona fide* sex-specific regulatory networks.

While the protocol we used for differentiation of ES cells into cardiac precursors has been derived from the extensive knowledge on cardiogenesis in vivo [41], the in vitro derivation of cardiac progenitors lacks other factors, such as spatial context, that are important for proper organ formation. For example, during heart development in vivo, multiple cell types, including transient populations, interact in three dimensions and receive input from surrounding tissues. However, single-cell analyses of early cardiac stages have pinpointed that cardiac progenitors derived from ES cells have a transcriptome corresponding to 9.5 dpc single-cell cardiomyocytes[43], a stage in which fibroblasts are not yet apparent. Thus, differentiated ES cells serve as a close approximation of the early stages of heart development.

### Sex-biased gene expression exists at every stage during cardiac development

To determine whether the sex biases in differentiated ES cells are present in vivo, we inspected previously published data from specific stages during heart development. Single-cell assessment of transcriptional profiles in early stages of cardiogenesis has allowed detailed analysis of the step-wise specification of cardiac progenitors, but the available data are not stratified by sex. For each sample, we genotyped for sex and re-analyzed these data and observed sex-biased expression across all the available stages of heart development. We also observed short bursts of sex-biased expression of regulatory factors at single stages, raising the question of whether these are capable of encoding persistent dimorphisms. In addition, we show that some genes equalize their expression, while others become biased in the opposite direction, which raises important questions on the mechanisms by which these events occur.

We recognize several caveats in this study. First, compiling datasets from different reports presents challenges because of the different experimental designs. Our own data is from ES cells in culture subjected to a directed differentiation protocol that only partially recapitulates the complex processes in vivo. Second, the single-cell RNA-seq data from embryonic and neonatal hearts, while useful for distinguishing cell populations, is necessarily incomplete. Currently, single-cell RNA-seq only detects a fraction of the transcriptome, with a bias towards high expression transcripts, which excludes many TFs that are expressed at relatively low levels.

Systems-level analyses have yielded valuable information on the correlations between congenital heart disease and their developmental origins [62, 63]. Transcriptomic data for early developmental stages is sparse, however. Nevertheless, the currently available datasets reveal sex-biased expression at every stage and suggest novel hypotheses for future mechanistic studies. Our analyses also open questions on how the fluctuations in sex-biased expression are regulated, how they are reflected in epigenetic differences between male and female cells, and how widely these occur in other tissues during embryogenesis. Our data also serves as a platform to identify the role of sex hormones in countering or compounding sex biases. Future studies will enable dissection of the effects of sex chromosomes and hormonal influence on sexual dimorphism. Ultimately, expanding developmental studies will allow us to connect early sexual dimorphism to the sex biases that occur in adult health and disease.

## Conclusions

The ability to profile transcriptomes has heightened interest in sex-biased gene expression, especially after recent reports that show substantial differences between males and females in humans and other animal models, even in organ systems that are overtly identical [4, 5, 44, 64]. The focus on adult tissues reflects a broadly held assumption that sex-biased expression is unimportant during early embryogenesis, during which critical lineage decisions are made, and that sex-specific selection only operates after the reproductive interests of the sexes have diverged [65]. In non-mammalian species, however, there is evidence that sex biases at the transcriptomic level occur throughout development [17, 66]. Here, we address a major gap in developmental studies by detecting sex-biased expression during mouse cardiac development. Our data strongly suggest that some of the differences in transcriptomic profiles in adult hearts may be established epigenetically before the appearance of sex hormones. Our observations open the field to explore the timing and extent of sex-specific transcriptional and epigenetic profiles in other organ systems and their relevance to sexual dimorphisms in adult health and disease.

## Additional files

Additional file 1: **Table S1.** List of antibodies used for ChIP-seq (XLSX 9 kb)

Additional file 2: **Table S2.** Accession and metadata of all samples analyzed in this study (XLSX 10 kb)

Additional file 3: **Dataset S1.** Genes in blue/violet module eigengene (XLSX 29 kb)

Additional file 4: **Dataset S2.** Ingenuity Pathway Analysis Upstream Regulators (XLSX 9 kb)

Additional file 5: **Dataset S3.** Topology analysis for protein-protein interaction networks (XLSX 141 kb)

Additional file 6: **Figure S1.** Modularity in male and female ES cell protein-protein interaction networks (JPG 50 kb)

Additional file 7: **Dataset S4.** Gene ontology for protein-protein interaction networks (XLSX 15 kb)

Additional file 8: **Figure S2.** qPCR of markers before and after ES cell differentiation (JPG 38 kb)

Additional file 9: **Dataset S5.** Differentially expressed genes in male and female cardiac precursors (XLSX 10 kb)

Additional file 10: **Figure S3.** Male-enriched genes after differentiation of ES cells. UCSC browser screen shots with tracks denoting Prdm14 occupancy (track obtained from Ma et al.) (JPG 41 kb)

Additional file 11: **Dataset S6.** Sex Biases in Single Cell Data (XLSX 108 kb)

Additional file 12: **Figure S4.** Modularity in protein-protein interaction networks (JPG 114 kb)

Additional file 13: **Figure S5.** Differentially expressed genes in adult male and female hearts (JPG 113 kb)

Additional file 14: **Datasets S7 and S8.** Ingenuity Pathway Analysis for sex-biased genes in adult heart (XLSX 19 kb)

Additional file 15: **Dataset S9.** Sex-biased Genes Shared between Human and Mouse Adult Hearts (XLSX 13 kb)

## Data Availability

Data generated has been deposited in GEO: GSE90516. Data from other reports and their supplementary information files was also analyzed: Li G, Xu A, Sim S, Priest JR, Tian X, Khan T, Quertermous T, Zhou B, Tsao PS, Quake SR *et al*: **Transcriptomic** Profiling Maps Anatomically Patterned Subpopulations among Single Embryonic Cardiac Cells. *Dev Cell* 2016, 39(4):491-507. GSE76118 DeLaughter DM, Bick AG, Wakimoto H, McKean D, Gorham JM, Kathiriya IS, Hinson JT, Homsy J, Gray J, Pu W *et al*: Single-Cell Resolution of Temporal Gene Expression during Heart Development. *Dev Cell* 2016, 39(4):480-490. Obtained from the author. Li B, Qing T, Zhu J, Wen Z, Yu Y, Fukumura R, Zheng Y, Gondo Y, Shi L: A Comprehensive Mouse Transcriptomic BodyMap across 17 Tissues by RNA-seq. *Sci Rep* 2017, 7(1):4200. PRJNA375882

## Abbreviations

EREs: Epigenetic and remodeling enzymes
ES cells: Embryonic stem cells
FPKM: Fragments per kilobase of exon per million reads mapped
GS: Gene significance
LIF: Leukemia inhibitory factor
ME: Module eigengene
MS: Module significance
PPIs: Protein-protein interaction networks
TFs: Transcription factors
TO: Topology overlap
WGCNA: Weighted gene co-expression network analysis
XCI: X chromosome inactivation
