# Supplementary figures and images for: The developmental origins of sex-biased expression in cardiac development
Source: Biol Sex Differ. 2019 Sep 5;10:46. doi: 10.1186/s13293-019-0259-1 (PMC6727560; doi:10.1186/s13293-019-0259-1)

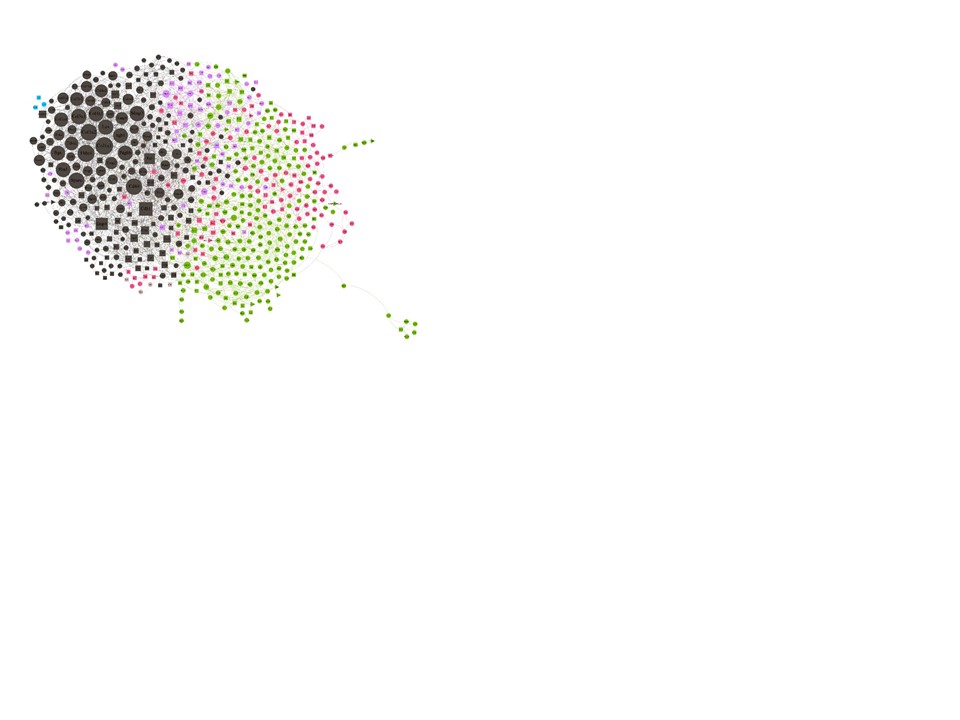

Supplement: Supplementary file 6 — Figure S1. Modularity in male and female ES cell protein-protein interaction networks (JPG 50 kb) [file 13293_2019_259_MOESM6_ESM.jpg]

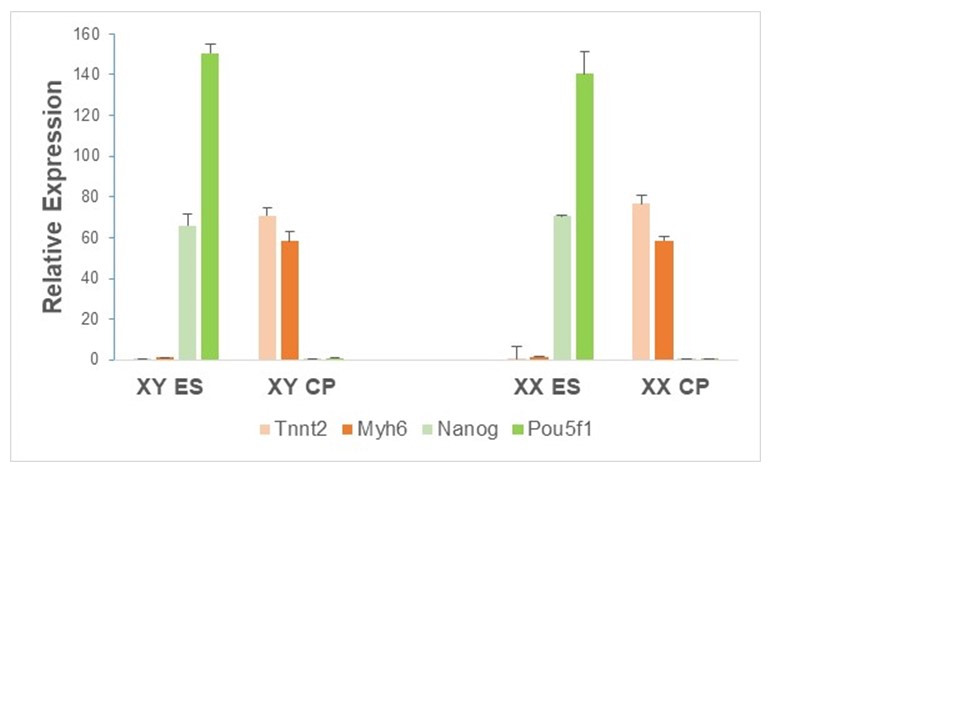

Supplement: Supplementary file 8 — Figure S2. qPCR of markers before and after ES cell differentiation (JPG 38 kb) [file 13293_2019_259_MOESM8_ESM.jpg]

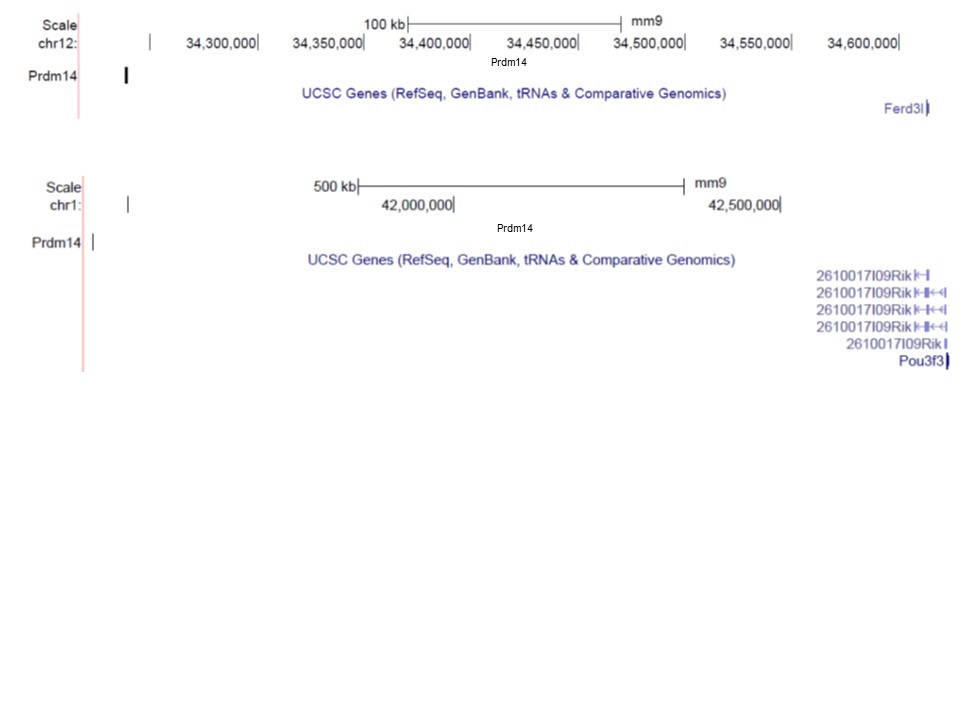

Supplement: Supplementary file 10 — Figure S3. Male-enriched genes after differentiation of ES cells. UCSC browser screen shots with tracks denoting Prdm14 occupancy (track obtained from Ma et al.) (JPG 41 kb) [file 13293_2019_259_MOESM10_ESM.jpg]

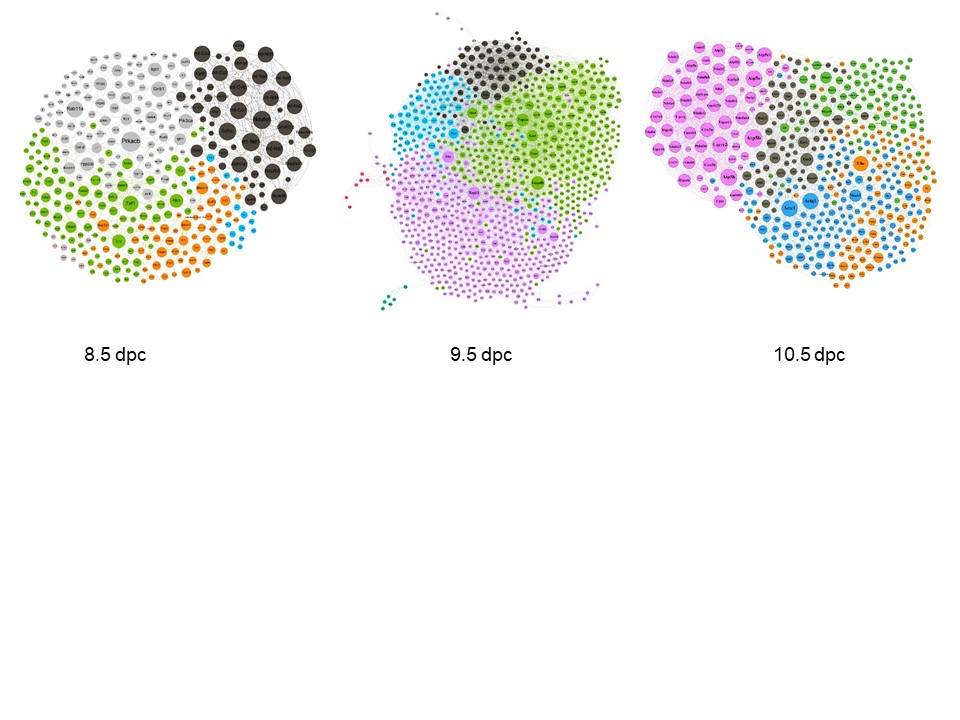

Supplement: Supplementary file 12 — Figure S4. Modularity in protein-protein interaction networks (JPG 114 kb) [file 13293_2019_259_MOESM12_ESM.jpg]

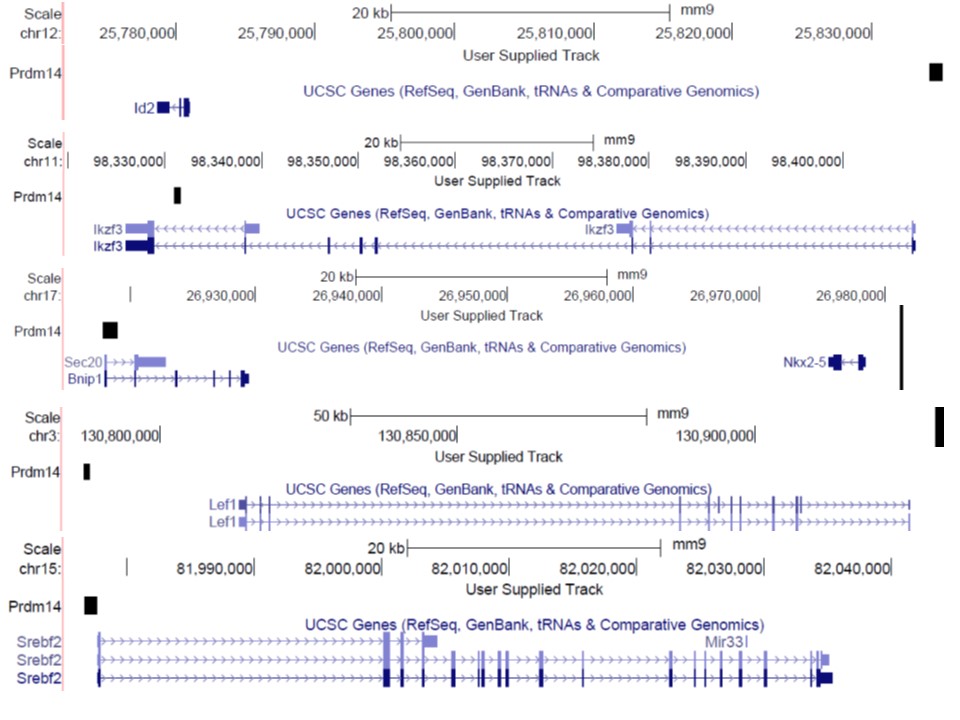

Supplement: Supplementary file 13 — Figure S5. Differentially expressed genes in adult male and female hearts (JPG 113 kb) [file 13293_2019_259_MOESM13_ESM.jpg]
